# Three Component Composite Scaffolds Based on PCL, Hydroxyapatite, and L-Lysine Obtained in TIPS-SL: Bioactive Material for Bone Tissue Engineering

**DOI:** 10.3390/ijms222413589

**Published:** 2021-12-18

**Authors:** Aleksandra Korbut, Marcin Włodarczyk, Karolina Rudnicka, Aleksandra Szwed, Przemysław Płociński, Monika Biernat, Paulina Tymowicz-Grzyb, Martyna Michalska, Natalia Karska, Sylwia Rodziewicz-Motowidło, Konrad Szustakiewicz

**Affiliations:** 1 Department of Polymer Engineering and Technology, Faculty of Chemistry, Wrocław University of Science and Technology (WUST), Wyb. Wyspiańskiego 27, 50-370 Wrocław, Poland; martynamichalska5656@gmail.com; 2 Department of Immunology and Infectious Biology, Faculty of Biology and Environmental Protection, University of Łódź, Banacha 12/16, 90-237 Łódź, Poland; marcin.wlodarczyk@biol.uni.lodz.pl (M.W.); karolina.rudnicka@biol.uni.lodz.pl (K.R.); aleksandra.szwed@biol.uni.lodz.pl (A.S.); przemyslaw.plocinski@biol.uni.lodz.pl (P.P.); 3 Biomaterials Research Group, Ceramic and Concrete Division in Warsaw, Łukasiewicz Research Network Institute of Ceramics and Building Materials, Postępu 9, 02-676 Warsaw, Poland; m.biernat@icimb.pl (M.B.); paulina.tymowicz@icimb.lukasiewicz.gov.pl (P.T.-G.); 4 Faculty of Chemistry, University of Gdańsk, Wita Stwosza 63, 80-308 Gdańsk, Poland; natalia.karska@ug.edu.pl (N.K.); s.rodziewicz-motowidlo@ug.edu.pl (S.R.-M.)

**Keywords:** polymers, foam scaffolds, PCL, cell adhesion, L-Lysine

## Abstract

In this research, we describe the properties of three-component composite foam scaffolds based on poly(ε-caprolactone) (PCL) as a matrix and hydroxyapatite whiskers (HAP) and L-Lysine as fillers (PCL/HAP/Lys with wt% ratio 50/48/2). The scaffolds were prepared using a thermally induced phase separation technique supported by salt leaching (TIPS-SL). All materials were precisely characterized: porosity, density, water uptake, wettability, DSC, and TGA measurements and compression tests were carried out. The microstructure of the obtained scaffolds was analyzed via SEM. It was found that the PCL/HAP/Lys scaffold has a 45% higher Young’s modulus and better wettability compared to the PCL/HAP system. At the same time, the porosity of the system was ~90%. The osteoblast hFOB 1.19 cell response was also investigated in osteogenic conditions (39 °C) and the cytokine release profile of interleukin (IL)-1β, IL-6, and tumor necrosis factor (TNF)-α was determined. Modification of PCL scaffolds with HAP and L-Lysine significantly improved the proliferation of pre-osteoblasts cultured on such materials.

## 1. Introduction

Tissue engineering and regenerative medicine are important fields in biomedical engineering. Progress in this area creates the need for novel scaffold materials and reproducible fabrication techniques [1]. Biopolymer-based materials are useful in regenerative medicine because they provide a temporary scaffold to help cells to form new bone tissue [2].

In recent years, aliphatic polyesters like polylactides, polyglycolide, polycaprolactone, and their copolymers have been widely investigated for biomedical applications [3]. The use of biocompatible and bioresorbable materials, with controllable degradation and resorption, enables optimal matching with the functional features of the tissue at the site of implantation [4]. One of the most promising materials in the field of bone tissue engineering is a composite containing apatite ceramics [5,6]. Usually, inorganic materials with a high content of calcium and phosphate are used (i.e., hydroxyapatite or tricalcium phosphate). Hydroxyapatite (HAP) is an inorganic material that forms the mineral phase of bone. The incorporation of mineral materials that have a similar chemical composition to mammals’ bones provides high biocompatibility and osteoconductivity. Recently, many articles have reported on the nanoscale formulation of HAP for orthopedics as a biomaterial to promote tissue regeneration [7,8,9]. Nanohydroxyapatite has proven advantageous due to its rapid resorption rate, which has been shown to result from its high solubility due to its nanocrystallinity [10].

Amongst biopolymers, poly(ε-caprolactone) (PCL) and its composites have been considered attractive candidates to produce scaffolds for tissue engineering applications. Their mechanical properties, thermoplastic processability, biodegradability, and biocompatibility towards bone within the human body have led to them being extensively used to design scaffolds for damaged bone regeneration [11]. PCL-based materials show sufficient porosity and appropriate pore size, which are required for enabling cellular ingrowth throughout the scaffold [12].

Many processing technologies have been reported to produce porous scaffolds by using biodegradable polymers, including 3D printing [6,13], porogen leaching [14], foaming with supercritical CO_2_ [15,16], electrospinning [17,18], and phase separation techniques [19,20]. Thermally induced phase separation (TIPS) enables the fabrication of scaffolds in the form of a foam. The porosity of scaffolds obtained using this technique is usually >90% [21]. The porosity and pore architecture (size and interconnectivity) of scaffolds have direct implications for their functionality during biomedical applications. A high degree of porosity promotes an increased proliferation rate. Furthermore, a porous surface improves the mechanical interlocking between the implant and the surrounding tissue, providing greater mechanical stability at this critical interface [16,22].

In this paper, we present three-component composite foam scaffolds based on poly(ε-caprolactone) as a matrix and hydroxyapatite whiskers (HAP) and L-Lysine as fillers, obtained using a combination of thermal induced phase separation and the salt leaching method (TIPS-SL). The ability of these scaffolds to support cell proliferation and differentiation was investigated. In particular, the effects of L-Lysine addition on the cell growth and mechanical properties of polymeric scaffolds were examined.

## 2. Results and Discussion

### 2.1. Apatite Properties

**Figure 1:**
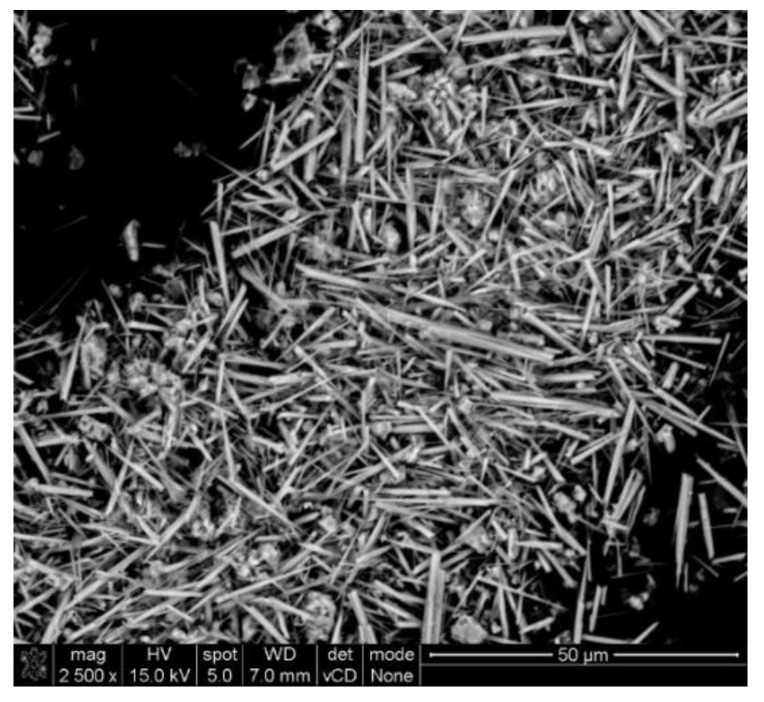
SEM image of multiphasic calcium phosphate whiskers.

Triphasic calcium phosphates were obtained and used in the work as reinforcing filler of the porous PCL composites. As can be seen in the SEM image (Figure 1), the obtained filler was in the well-defined whiskers form. According to the data, ceramic particles in whiskers form improve the mechanical strength of polymer composites [23]. The length and width of the obtained whiskers were within the range of 10–30 μm and 1–3 μm, respectively, and the length-to-width ratio of the obtained whiskers was approximately 16.7.

**Figure 2:**
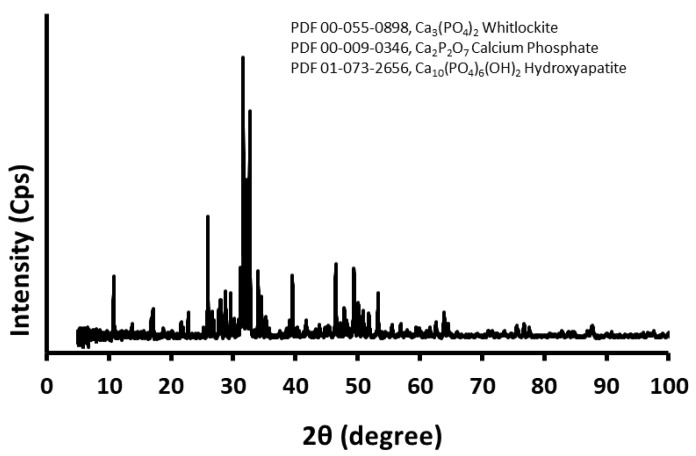
XRD pattern of the obtained triphasic calcium phosphate whiskers.

The whiskers also contained approximately 72% bioactive hydroxyapatite, which is more degradable, and whitlockite and calcium pyrophosphate in the amounts of approximately 15% and 12%, respectively (Figure 2) [24]. The assumption of the whiskers application was also that different degradation rates of individual phases in the whiskers could improve reinforcement and osteoconduction during a long PCL composite degradation.

### 2.2. Physical Properties of PCL-Based Scaffolds

**Figure 3:**
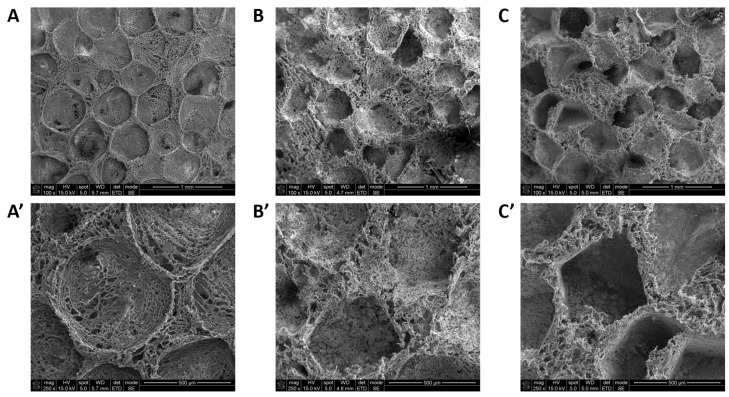
SEM images of scaffolds: (**A**,**A’**) 10% of PCL. (**B**,**B’**) 10% of PCL/HAP_50:50 *w*/*w*. (**C**,**C’**) 10% of PCL/HAP/Lys_50:48:2 *w*/*w*.

The internal pore structures of the samples were observed using FE-SEM microscopy. From this figure, it is possible to observe that all scaffolds (neat PCL and the composites) present a porous structure. SEM measurements (Figure 3) show scaffolds having two sizes of pores. One was bigger, with a diameter of up to 600 µm, and remained after the salt leaching process. The second type of pore was located in the walls of the micropores and had a diameter up to 50 µm. This was a result of the sublimation of 1,4-dioxane. In the case of composite samples (PCL/HAP and PCL/HAP/Lys), pore walls were evenly covered by apatite whiskers. Compared to the PCL scaffold, the pore wall surfaces of the PCL/HAP or PCL/HAP/Lys scaffolds were rougher than those of PCL scaffolds because of the incorporation of HAP whiskers.

As listed in Table 1, the obtained scaffolds exhibited a high porosity, more than 90%. For the neat PCL foams, porosity is on the level of 93.6%. Adding apatite whiskers or/and L-Lysine as filler resulted in a slight decrease in porosity (~2–3%) compared to neat PCL. On the other hand, the density of the composite scaffolds was higher than that of neat PCL foams. This was caused by the high density of hydroxyapatite (HA) compared to PCL.

The mechanical properties of the foams were investigated by a compression test. The compressive stress and Young’s modulus of the PCL-based foams are listed in Table 2. The values of Young’s modulus ranged from 204.6 to 464.4 kPa and significantly increased for composite scaffolds. The highest values were observed for foams that contained additional L-Lysine.

The static contact angle (CA) and water uptake were also measured. The results are shown in Table 3. The wettability measurements of scaffolds represemt an important factor that affects the interaction with cells [25]. PCL is a hydrophobic material, whereas hydroxyapatite is known to be a hydrophilic material with a water contact angle of ~10°. The wettability of PCL-based composites improved, and the water contact angle decreased from 87.8 ± 3.0 for neat PCL to 77.9 ± 4.5 for PCL/HAP and 72.6 ± 4.2 for PCL/HAP/Lys. The results of the CA measurements indicated that the layer of hydroxyapatite in the pore walls significantly improved the wettability of foams. Moreover, the percentage of water absorption in the obtained foams was calculated. For both types of PCL foam composites, the results obtained indicated that moisture absorption decreased with the addition of a filler.

### 2.3. Thermogravimetric Analysis

**Figure 4:**
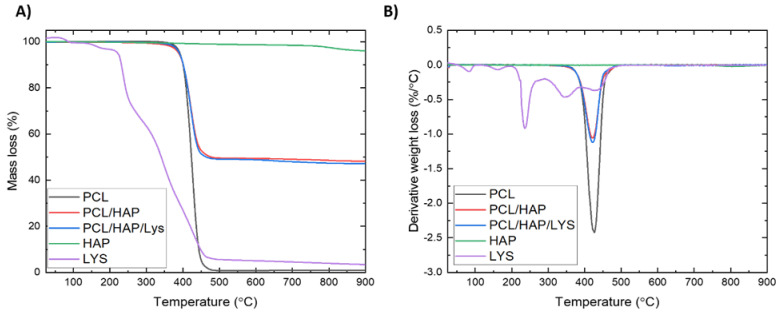
(**A**) Thermogravimetric curves of PCL-based foams. (**B**) First derivative of the TGA curves of PCL-based foams.

Based on the TGA analysis, the hydroxyapatite and L-Lysine content in composite foams was determined from the difference in mass loss at 900 °C. TGA and DTG curves are presented in Figure 4. The mass loss at 900 °C of foams without and contain L-Lysine was 51.81% and 52.84%, respectively. The TGA results (Table 4) indicated good agreement between the estimated and processing filler contents. Furthermore, to investigate the influence of fillers on the thermal stability of foams, the temperature corresponding to a 5 wt % loss of mass (T_-5wt.%_) was defined. TGA analysis revealed that the thermal stability of a composite that contains L-Lysine is similar to that of neat PCL foams, while the T_-5wt.%_ value for a composite without L-Lysine is 3.4 °C lower than that of neat PCL.

### 2.4. Thermal Properties of PCL-Based Scaffolds

**Figure 5:**
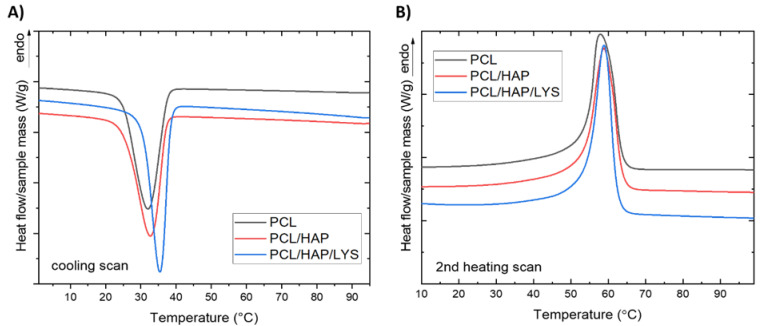
(**A**) The cooling DSC curves of PCL-based foams. (**B**) The second heating DSC curves of PCL-based foams.

The composite samples were first heated to 100 °C and maintained at this temperature for 5 min under nitrogen to erase the previous thermal history. The cooling and second heating DSC curves are shown in Figure 5. The values of estimated thermal parameters are presented in Table 5. In all the DSC curves, two characteristic thermal effects are observed: crystallization at ~32.5–35.6 °C, and melting at temperatures over 53 °C. During the cooling from the melt, PCL underwent crystallization with an onset at 37.3 °C. For neat PCL foams and the PCL/HAP and PCL/HAP/Lys composites, the exotherm of melt crystallization is visible. The enthalpy of melt crystallization (ΔH_c_) occurring with a cooling rate of 10 °C/min had the highest value for neat PCL (55.8 J/g), while in composites the enthalpy was lower (−27.3 J/g for both samples). The temperature of melt crystallization was slightly higher for both composites than for the neat PCL foam. These results indicate an influence of the HAP content on the nucleating activity.

On second heating, DSC curves’ endothermic melting peaks are visible. The results show that the neat PCL melting temperature is 58.3 °C; this value is about 0.9 °C lower than for composite foams. The crystallinity degree (X_c_) of neat PCL foam and composite foams was determined using Equation (3) and the results are shown in Table 5. All samples presented a similar crystallinity of about 40%.

### 2.5. L-Lysine Release

**Figure 6:**
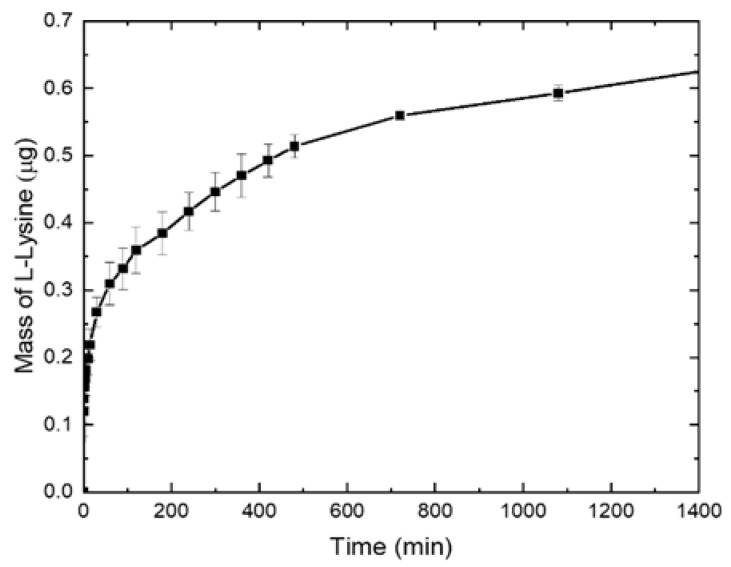
L-Lysine release from the PCL/HAP/Lys composite over 1440 min. The mass of lysine released was converted into a 50-mg disc of composite.

In this study, we present the simplest strategy for L-Lysine incorporation into a porous scaffold using the suspension of HAP/L-Lysine in a PCL solution and the TIPS-SL technique. The last step in the scaffold preparation process includes leaching the salt, which is associated with the risk of washing lysine from the scaffold. For this reason, the level of lysine released from the composite scaffold was verified. L-Lysine incorporated in the composite showed a rapid initial release from the scaffold, within 100 min. After 800 min, the level of lysine remained almost unchanged. The kinetics profile of L-Lysine release is presented in Figure 6. The HPLC results show that lysine is still contained in the scaffold.

### 2.6. Cytocompatibility and Osteoconductivity

#### 2.6.1. Cell Colonization of PCL-Based Scaffolds

**Figure 7:**
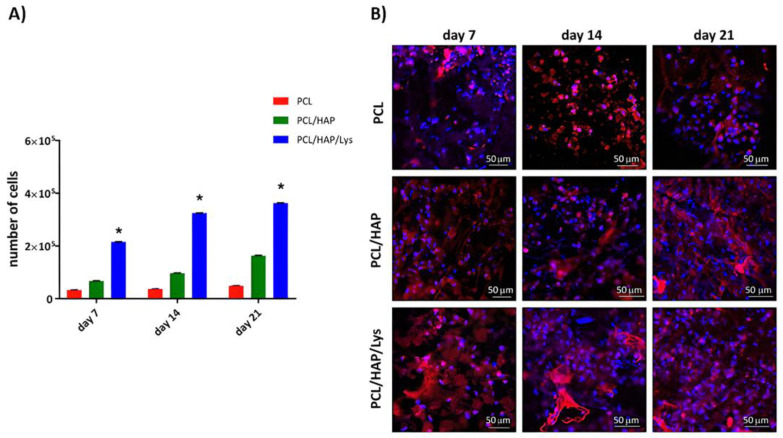
(**A**) The proliferation of human osteoblasts cultured on PCL-based composites under osteogenic conditions (39 °C) for up to 21 days. The results represent the mean values ± SEM. * *p* < 0.05 between the PCL and PCL-modified composites, based on the results of a one-way ANOVA (Kruskal–Wallis test) evaluation. (**B**) Cell colonization of scaffolds: morphology and adhesion of osteoblasts visualized by confocal laser scanning macroscopy (Leica TCS LSI) after 7, 14, and 21 days of incubation with tested PCL, PCL/HAP, and PCL/HAP/Lys composites. Cells colonizing the PCL foams on the surface were stained with Texas Red-phalloidin (red, F-actin) and 4′,6-diamidino-2-phenylindole (DAPI) (blue, nuclei). Each panel represents 2D pictures of foam scaffolds. Our results indicate that the addition of L-Lysine to the scaffold has a similar positive effect on cell proliferation as the addition of poly-Lysine. These results are in line with findings of Wang et al., who showed that modification of the PCL surface with poly-Lysine, collagen, and HAP promoted the growth and differentiation of bone-marrow-derived stromal cells (BMSCs) [26]. These findings are also in line with the results reported by Li et al., who observed that mineralized gelatin-coated PCL scaffolds provide a favorable microenvironment for the growth and proliferation of MC3T3 cells [27]. Interestingly, PCL scaffolds have been developed to mimic the bone environment, but also as a drug delivery tool for therapeutic agents, such as dexamethasone (DXM), as described by Palamà et al. [28]. MG63 osteoblasts cultured on PCL scaffolds without loaded DXM revealed slower proliferation activity as compared to the osteoblasts cultured on DXM-loaded scaffolds.

The dynamics of tissue regeneration is highly dependent on the first stages of the composite colonization by target cells, and their readiness to adhere and propagate within the biomaterial. In an ideal strategy, mesenchymal stem cells are attracted to colonize the scaffold and can then differentiate into mature osteoblasts, which drive and coordinate future bone regeneration. In this study, we used hFOB1.19 cells, model pre-osteoblasts with many features of mesenchymal stem cells, which are able to terminally differentiate into proper osteoblasts. Initially, we evaluated the morphology of hFOB 1.19 osteoblasts and their ability to adhere and colonize the PCL-based composites (Figure 7). To address the question of whether the colonization of the composites is accompanied by their proliferation or differentiation into osteogenic cells, the osteoblasts were incubated at 39 °C with PCL-based composites and their proliferation rate and morphology were evaluated over the course of 21 days (data collected on days 7, 14, and 21). We have shown that PCL/HAP/Lys scaffolds, in comparison to PCL/HAP and PCL scaffolds, promote efficient pre-osteoblast proliferation (Figure 7A). The mean number of cells (MNoC) identified within PCL/HAP/Lys scaffolds was significantly (*p* < 0.05) higher as compared to the proliferation rate induced by the reference (PCL) composite. The MNoC identified within PCL/HAP/Lys reached 2.2 × 10^5^ ± 2.1 × 10^2^ (on day 7), 3.3 × 10^5^ ± 6.5 × 10^2^ (on day 14), and 3.6 × 10^5^ ± 3.0 × 10^2^ (on day 21) and were significantly higher as compared to MNoC for the control (PCL) composite: 0.3 × 10^5^ ± 3.2 × 10^2^, *p* = 0.004 (on day 7), 0.3 × 10^5^ ± 6.3 × 10^2^, *p* = 0.004 (on day 14) and 0.4 × 10^5^ ± 6.7 × 10^2^, *p* = 0.004 (on day 21). In addition, osteoblasts cultured with PCL/HAP exhibited a higher proliferation rate, compared to the PCL scaffold. We have also shown that PCL/HAP/Lys-dependent proliferation was accompanied by successful colonization of the composite by osteoblasts (Figure 7B), which is evidenced by the higher cell densities observed after 7, 14, and 21 days of incubation with PCL/HAP and PCL/HAP/Lys composites.

#### 2.6.2. Release Profile of Immunomodulatory Cytokines

Bone remodeling is controlled by molecules of the immune system, including cytokines, receptors, signaling molecules, and transcription factors. The major role of cytokines is the regulation of cell growth and differentiation and cell survival, as well as the control of gene expression. To check the cytokine release profile of the newly developed PCL-modified scaffold, hFOB1.19 cells were cultured on the surface of the biomaterial for up to 21 days and the levels of IL-1β, IL-6, and TNF-α in the cell culture supernatants were evaluated.

**Figure 8:**
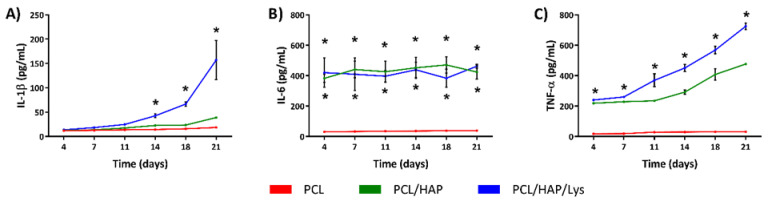
Quantification of the immunomodulatory cytokines (**A**) IL-1β, (**B**) IL-6, and (**C**) TNF-α assayed in the supernatants of hFOB 1.19 cultured on PCL scaffolds at osteoinductive conditions (39 °C). The results represent the mean values ± SEM. * *p* < 0.05 between the PCL and PCL-modified composites, based on the results of one-way ANOVA (Kruskal–Wallis test) evaluation. Our findings suggest that the modification of a foam scaffold with HAP and L-Lys enhances IL-1β, IL-6, and TNF-α production by hFOB 1.19 cells, whereas the PCL/HAP composite promotes significant IL-6 and moderate TNF-α production. Inflammation constitutes the first physiological phase of the bone tissue regeneration process. The involvement of proinflammatory cytokines during the first days after implantation is necessary to attract and activate immune cells, which clear damaged tissue and dead cells from the site of injury. On the other hand, IL-6, IL-1β, and TNF-α are known to be strong proinflammatory cytokines that control both osteoclast and osteoblast differentiation [29]. IL-6 enhances the differentiation of osteoblasts’ precursors and protects osteoblasts from apoptosis [30,31]. Recent findings indicate that IL-1β is the key cytokine triggering the osteoblastic transition of other cell types and is the primary signal for tissue calcification [32]. Thus, elevated production of IL-1β, IL-6, and TNF-α in response to PCL/HAP/Lys (and partially by the PCL/HAP scaffold) facilitates the differentiation of mesenchymal stem cells into osteoblastsprocesses that are important for bone remodeling [33]. These results are in line with our previous observations in PGS/HAP biomaterials. The addition of HAP had a similar effect on the hFOB1.19 cytokine release profile, favoring the terminal differentiation of osteoblasts. While the signal for IL-6 production was then evident for cells cultured on each material containing HAP, the production of IL-1β was only apparent when cells were cultured under osteoinductive conditions, and strongly correlated with further signs of efficient calcification [34].

We have shown that the modification of foam scaffolds with HAP and Lys led to a significant increase in IL-1β, IL-6, and TNF-α production by hFOB 1.19 as compared to the concentration of these cytokines detected in cell cultures incubated with PCL (Figure 8A–C). The increase in IL-1β induced by PCL/HAP/Lys in comparison to PCL scaffolds was especially noticeable from day 14 (42.4 ± 3.7 pg/mL and 14.2 ± 0.8 pg/ mL, *p* = 0.005), through day 18 (66.6 ± 4.7 pg/ mL and 15.8 ± 0.2 pg/ mL, *p* = 0.004) and at the end of the experiment (157.1 ± 40.1 pg/ mL and 18.4 ± 0.5 pg/ mL, *p* = 0.004) (Figure 8A).

PCL/HAP and PCL/HAP/Lys scaffolds induced significantly and dramatically higher levels of IL-6 as compared to basic composites (PCL), and the production of this mediator occurred as early as on day 4 of culture and remained at a similar level throughout the whole period of the assay (Figure 8B). Moreover, the production of TNF-α was significantly higher in hFOB 1.19 cell cultures incubated with PCL composites modified with HAP and L-Lys at all time points as compared to the level of TNF-α detected in cell cultures exposed to PCL alone, e.g., 239.9 ± 6.0 pg/m mL and 17.9 ± 2.6 pg/m mL *p* = 0.0003 (on day 4) and 724.9 ± 21.5 pg/m mL and 30.9 ± 1.1 pg/m mL, *p* = 0.004 (on day 21), respectively (Figure 8C).

#### 2.6.3. Alkaline Phosphatase

**Figure 9:**
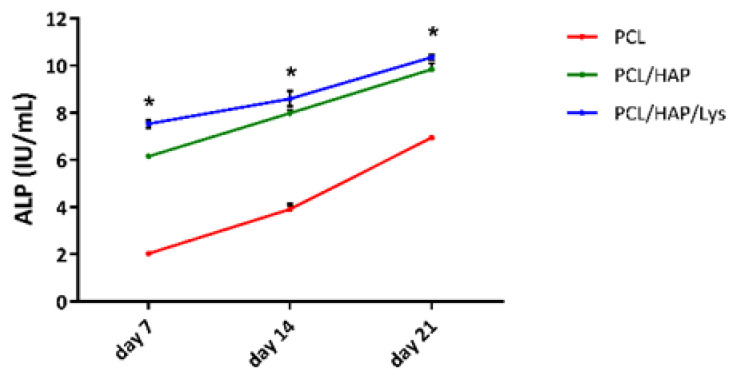
Alkaline phosphatase (ALP) activity in cell lysates obtained from hFOB 1.19 osteoblasts cultured in the osteoinductive environment at 39 °C. The results represent the mean ALP concentration ± SD. * *p* < 0.05 between the PCL and PCL-modified composites, based on the results of a one-way ANOVA (Kruskal–Wallis test) evaluation.In our study, the levels of ALP produced by osteoblasts cultured on the PCL-modified scaffolds were higher than the ALP concentrations detected in cell cultures exposed to PCL composites. Our results are corroborated by the findings reported by Shitole et al. [35]. They observed that PCL nanofibers modified with nano-hydroxyapatite (nHAP) enhance the ALP activity of MG-63 cells and result in a better mineralization capacity as compared to pure PCL. It is worth emphasizing that, in our research, the modification of the PCL/HAP scaffold with L-Lysine resulted in the intensification of ALP production. This might be a result of more cationic sites on the lysine-coated surface of the PCL/HAP scaffolds. The higher availability of cationic sites causes increased adhesion and further differentiation, and thus an increase in the ALP level [36].

Alkaline phosphatase (ALP) activity serves as an early marker for the osteoblast differentiation involved in bone calcification and hard tissue formation. Osteoblastic differentiation was assessed by measuring ALP activity after 7, 14, and 21 days of hFOB 1.19 osteoblasts cocultured with the PCL-based foam scaffolds. We have observed the upregulation of ALP production in cell cultures induced by PLA/HAP/Lys and PLA/HAP scaffolds, whereas no such effect was observed for cells cultured with control (PCL) scaffolds. The PCL/HAP scaffold was modified with L-Lysine-stimulated hFOB 1.19 cells to produce significantly higher ALP concentrations than those detected in cell cultures exposed to PCL composites on days 7, 14, and 21 (7.5 ± 0.2 U/mL, *p* = 0.02, 8.6 ± 0.3 U/mL, *p* = 0.02, 10.4 ± 0.1 U/mL, *p* = 0.02, respectively) (Figure 9). Although PCL/HAP also induced an increase in ALP activity, these differences were not recognized as statistically significant: the level of ALP produced by osteoblasts cultured on the PCL and PCL/HAP scaffolds were as follows (2.0 ± 0.08 U/mL and 6.1 ± 0.02 U/mL on day 7; 3.9 ± 0.2 U/mL and 7.9 ± 0.2 U/mL on day 14; and 6.9 ± 0.1 U/mL and 9.8 ± 0.2 U/mL on day 21, respectively).

## 3. Materials and Methods

### 3.1. Materials

The poly(ε-caprolactone) used in this research work was CAPA 6800 (high-molecular-weight linear polyester, Mw: ~80,000 g/mol), purchased from Perstorp (Malmö, Sweden). Apatite whiskers (HAP) obtained as multiphasic calcium phosphate were prepared using a methodology described earlier [9,24,36]. L-Lysine (crystallized, >98%) was purchased from Sigma-Aldrich (St. Louis, MO, USA). Sodium chloride and 1,4-dioxane were supplied by POCH (Gliwice, Poland). The standards of L-Lysine, hydrochloride, ammonia formate, formic acid, and acetonitrile (HPLC-grade) were purchased from Sigma-Aldrich. All aqueous solutions were prepared using Milli-Q water (Millipore, Bedford, MA, USA).

### 3.2. PCL Foam Scaffold Preparation

PCL (as the reference scaffold sample) and PCL-based composite scaffolds (PCL/HAP and PCL/HAP/lys) were produced using a thermally induced phase separation technique supported by a salt leaching process (TIPS-SL). Designation of the samples are given in Table 6. In the first step, PCL (10% *w*/*w*) was dissolved in 1,4-dioxane under stirring conditions for 24 h to obtain a homogeneous solution. After this time, the required amounts of HAP or HAP/Lys were suspended in the polymer solution and sonicated for 30 min (at 60 °C) in order to obtain a homogeneous suspension. Then, 1.5 g of NaCl and 0.75 mL of the polymer solution/suspension were added to 24-well plates. All scaffolds were produced using NaCl salt having a size of 500–600 µm. The 24-well plates were placed in a freezer for 24 h. Frozen samples were transferred to a freeze-drying vessel and submitted to a solvent removal process (at −80 °C and vacuum ∼10–20 Pa for 24 h). Dried samples were then taken out and put in demineralized water to dissolve the salt and remove it from the scaffolds. The salt leaching process was supported by slow mixing on a magnetic stirrer. Water (in a 5 L beaker) was changed three times over 24 h. After leaching, the scaffolds were dried at 60 °C for 24 h.

### 3.3. Scanning Electron Microscopy (FE-SEM)

The microstructure of the obtained porous composites was tested by scanning electron microscopy with field emission (Nova NanoSEM 200, FEI, Eindhoven, Holand). Before the study, the samples were covered with a conductive material (25 nm gold film) using a sputter coater (EM SCD500, Leica, Vienna, Austria). Imaging of composites was visualized in high vacuum conditions using an ETD detector (Everhart–Thornley detector combined with Nova NanoSEM 200) at 15 kV accelerating voltage and at magnifications of 100× and 250×.

### 3.4. Compressive Strength

The compressive strength of the foams was assessed using a Universal testing machine (INSTRON 5960, Norwood, MA, USA) fitted with a 1 kN head. The compression speed was set at 2 mm/min. The barrel-shaped samples were cut into specimens 4 mm high and with a diameter of ~15 mm. The samples were tested to obtain a maximum strain of 90%. At least 10 samples of each kind were measured. The compressive stress and Young’s modulus were determined at a strain in the range between 5% and 90%.

### 3.5. Density Measurements

The porosity of the scaffolds was calculated using the following Equation (1) [22]:

(1) $$\Phi_{p}=\left[1-\frac{\rho_{sc}}{\rho_{b}}\right]\;$$

where *Φ_p_* is the porosity, *ρ_sc_* is the scaffold density obtained from the buoyancy method using a Hildebrand Electronic Densimeter H-300S (Wendlingen am Neckar, Germany), and *ρ_b_* is the bulk density of the composite scaffold (also measured using H-300S). At least 10 samples of each kind were measured.

### 3.6. Wettability Measurements

Water surface tension measurements were conducted using a PG-X contact angle goniometer (Testing Machines, Inc., New Castle, DE, USA). Each measurement was repeated at least 10 times for each sample and the average value as well as the standard deviation were calculated.

### 3.7. Water Uptake Measurements

The water absorption of porous materials refers to their ability to absorb water and is defined as the ratio of the mass of the wet sample to the mass of the dry sample, as Equation (2) [37]:

(2) $$W.U.=\;\frac{m_{w}-m_{d}}{m_{d}}\cdot 100\%$$

where *W.U.* is the water uptake (%), *m_w_* is the mass of wet foam, and *m_d_* is the mass of dry foam (the weight of the foam in the beginning of the test) (g). Dry foams were weighed and then soaked with demineralized water for 10 min. After this time, samples were dried inside the vacuum dryer (600 hPa, 20 °C, 20 min). The experiment was repeated three times for each foam.

### 3.8. Thermogravimetry (TGA)

The thermogravimetric analysis was performed with a TGA/DSC1 Mettler Toledo thermobalance (Columbus, OH, USA). Samples were heated at a rate of 10 °C/min from 25 °C to 900 °C under a 30 mL/min air flow. For the data presentation, the TGA curves were exported to OriginPro 2021 (OriginLab, Northampton, MA, USA) as ASCII files.

### 3.9. Differential Scanning Calorimetry (DSC)

Samples were analyzed on the Mettler Toledo DSC1 system, coupled with a Huber TC 100 intracooler (Offenburg, Germany), under the following conditions: mass: ~5.5 mg; nitrogen flow: 60 mL/min; heating or cooling rate: 10 °C/min, temperature range: 0 °C to 100 °C. After the heating cycle, the samples were thermally equilibrated at 100 °C for 5 min and cooled down to 0 °C. A second heating scan was also performed. The crystallinity degree (Xc) of PCL in the materials was determined from the melting peak area of second heating according to the following Equation (3) [38]:

(3) $$X_{c}=\frac{\Delta H_{m}}{w\cdot \Delta H_{m}^{100\%}}\cdot 100\%$$

where Δ*H_m_* is the melting enthalpy [J/g], *w* is the weight fraction of PCL in composite foams, and Δ*H_m_*^100%^ is the melting enthalpy of 100% crystalline PCL (139 J/g) [38]. Experimental data were processed using the generic STARe computer program (Mettler Toledo, Columbus, OH, USA). For data presentation, the DSC profiles were exported to OriginPro 2021 as ASCII files.

### 3.10. L-Lysine Release

In order to establish a calibration curve for lysine, three identical solutions of lysine were prepared, for which chromatographic analyses (HPLC) were performed. A standard lysine solution (1 mg/mL) was prepared by dissolving the amino acid (L-Lysine hydrochloride) in a mixture of mobile phase solution A/water, 1:1 (*v*/*v*), where A is 5 mM formic acid and 5 mM ammonium formate in an acetonitrile/water (9:1) solution. Twelve dilution points have been designated. For the obtained results, the relationship between the area under the peak in the HPLC chromatogram and the concentration was determined using Shimadzu LCsolution software (Shimadzu Co., Kyoto, Japan). The results of three separate measurements were averaged.

The composite foam (with lysine; without as a control) was weighed and placed in Eppendorf tubes, to which 1 mL of water was added. The composite foam was incubated at 37 °C with gentle shaking (400 rpm) for 24 h. The lysine release from the composite was tested at the following time points: 0, 1, 2, 3, 4, 5, 10, 15, 30, 60, 90, 120, 180, 240, 300, 360, 420, 480, 720, 1080, and 1440 min. The analysis of the released lysine was performed using a NEXERA X2 chromatography system (Shimadzu, Duisburg, Germany) consisting of two pumps (LC 30AD), a ELSD LTII detector, an SPD-M20A detector, an automatic dispenser (SIL 30AC), a column thermostat (CTO-20AC), and a degasser membrane (DGU-20A5R). HPLC analysis was performed with a linear 0–100% B gradient for 30 min on a bioZen™ (Phenomenex Inc., Torrance, CA, USA) 2.65 µm Glycan (2.1 mm × 250 mm) column, where A: 5 mM ammonium formate and 5 mM formic acid in an acetonitrile/water mixture of 9:1 (*v*/*v*); B: 5 mM ammonium formate and 5 mM formic acid in a acetonitrile/water mixture of 7.5:2.5 (*v*/*v*), pH 7.5. The volumetric flow rate was 0.25 mL/min. Peaks were detected at a wavelength of 223 nm. The injection volume was 50 µL for each sample. The mass of lysine released was calculated on the basis of the value of the peak area of the released lysine and the previously determined calibration curve for lysine.

### 3.11. Bioefficacy of the PCL-Based Foam Scaffolds

Considering the future utility of the designed foam scaffolds as biomaterials supporting bone regeneration, it is necessary to explore their influence on osteoblasts in regard to metabolic activity/viability, proliferation, and morphology.

#### 3.11.1. Sterilization of PCL-Based Foam Scaffolds

Prior to biological evaluation, PCL-based foam composites were sterilized by gamma irradiation with a dose of 35 kGy with gamma rays in a ^60^Co source at the Institute of Applied Radiation Chemistry in Lodz. The effectiveness of the sterilization process was confirmed on microbiological media intended for bacteria and yeast growth.

#### 3.11.2. Cell Culture and Propagation

CRL-11372™, the human fetal osteoblastic cell line (hFOB 1.19) used in this study, was obtained from the American Type Culture Collection (ATCC, Manassas, VA, USA). The cells were cultured in a 1:1 mixture of phenol-free Dulbecco’s modified Eagle’s medium and Ham’s 12-F medium (Gibco, Thermo Fisher Scientific, Waltham, MA, USA). Media were supplemented with 2.5 mL of L-Glutamine (100X), 10% heat-inactivated fetal bovine serum (FBS) (HyClone, Cytiva, Marlborough, MA, USA), and 0.3 mg/mL of geneticin (G418) (Sigma-Aldrich). Cells were cultured at 34 °C, in a humidified atmosphere containing 5% CO_2_. Cell cultures were observed using an inverted microscope (Motic AE2000, Xiamen, China) to ensure that they form confluent and homogeneous monolayers. The medium was changed every three days under aseptic conditions. Passaging of confluent cell monolayers was carried out at 80–90% confluence using a 0.5% trypsin–0.05 mM EDTA solution (Gibco).

#### 3.11.3. Osteoconductivity Assay

**Figure 10:**
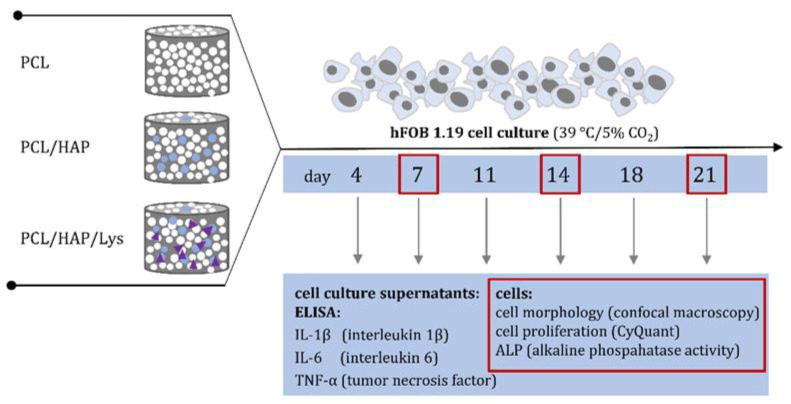
The timeline for biological studies of PCL, PCL/HAP, and PCL/HAP/Lys scaffolds based on cell analysis or soluble markers of osteoinduction evaluated at selected time points.

hFOB 1.19 osteoblasts were seeded on PCL-based foam composites as described previously [9,39]. Briefly, foam scaffolds were plated into 24-well culture plates (Nunclon Sphera, Nunc, Thermo Fisher Scientific, Waltham, MA, USA) and stabilized with VetGlue (SuperVet Glue, EVi-MED, Liszki, Poland). The scaffolds were soaked with 200 µL of osteogenic medium for osteoblasts and incubated at 39 °C and 5% CO_2_ for 2 h. Following the initial soaking, 20 µL of fresh cell suspension (5 × 10^5^ cells) was added to the center of each scaffold. To induce proliferation and encourage differentiation, composites with hFOBs were cultured in an osteogenic medium (supplemented with 1% heat-inactivated fetal bovine serum (HyClone, Cytiva), 0.3 mg/mL geneticin, 50 μg/mL ascorbate-2-phosphate (Sigma-Aldrich, Saint Louis, MO, USA), 1 μM dexamethasone (Sigma-Aldrich), and 10 mM β-glycerophosphate (Sigma-Aldrich) at 39 °C. The medium was changed every third day for 21 days. All supernatants collected on days 4, 7, 14, 18, and 21 were stored at −80 °C until further evaluation of the soluble markers (see Section 3.11.4) and cell lysates collected on days 7, 14, and 21 were used for proliferation analysis (see Section 3.11.5) and ALP quantification (see Section 3.11.6) (Figure 10).

#### 3.11.4. Visualization of Cell Adhesion

To visualize osteoblasts within PCL-based foam composites, on days 7, 14, and 21 the scaffolds were washed with PBS and fixed with a 3.7% formaldehyde (Sigma-Aldrich) solution for 20 min at room temperature. Following permeabilization (15 min, 0.1% Triton X-100 in PBS) the cells were counterstained with phalloidin conjugated with Texas Red^®^-X (Thermo Fisher Scientific) and DAPI (Thermo Fisher Scientific) to visualize cytoskeletal F-actin filaments and the nucleus, respectively. The scaffolds were observed with a confocal macroscope (Leica TCS LSI, Leica Microsystems, Frankfurt, Germany) with objective 5×/0.50 LWD and imaged with the following fluorescence conditions: Texas Red^®^-X (Ex_596nm_/Em_615nm_) and DAPI (Ex_360nm_/Em_460nm_). Leica Application Suite X software (LAS X, Leica Microsystems) was used for cell imaging. Confocal analysis was performed in the Laboratory of Microscopic Imaging and Specialized Biological Techniques at the Faculty of Biology and Environmental Protection at the University of Lodz, Poland.

#### 3.11.5. Cell Proliferation Assay

The proliferation of osteoblasts was evaluated after 7, 14, and 21 days of incubation with PCL-based foam scaffolds using CyQUANT Cell Proliferation Assay (Invitrogen, Thermo Fisher Scientific). At the selected time points, cells within the foam composites were washed with PBS and frozen at −80 °C. At the time of the assay, samples were thawed at room temperature and lysed in a buffer containing CyQUANT-GR dye, prepared according to the manufacturer’s instructions. Fluorescence signals (excitation 405 nm, emission 520 nm) were detected using a SpectraMax^®^ i3x Multi-Mode Microplate Reader (Molecular Devices, San Jose, CA, USA).

#### 3.11.6. Determination of the Cytokine Release Profile

The concentrations of IL-1β, IL-6, and TNF-α were determined using specific sandwich ELISA kits (R&D Systems, Minneapolis, MN, USA) in accordance with the manufacturer’s instructions. The minimum detectable levels were 3.91 pg/mL for IL-1β, 9.38 pg/mL for IL-6, and 15.6 pg/mL for TNF-α. In brief, wells on a 96-well half area plate (Greiner Bio-One, Greiner Bio-One GmbH, Kremsmünster, Austria) were coated overnight at room temperature with capture antibodies in PBS, then washed in PBS/0.05% Tween 20 and blocked with PBS containing 1% BSA for 1 h. After washing the wells with PBS/0.05% Tween 20, samples were added and incubated for 2 h. Standard curves were also established using serial dilutions of known concentrations of recombinant human cytokines. Afterwards, wells were washed with PBS/0.05% Tween 20 and incubated for 2 h with biotinylated monoclonal antibody diluted 1:60 in PBS/1% BSA. Following washing, a streptavidin–horseradish peroxidase (SaV-HRP) solution (diluted 1:40) was added and incubated for 20 min. After washing the wells with PBS/0.05% Tween 20, a mixture of tetramethylbenzidine (TMB) and hydrogen peroxide was added. The colorimetric reaction was stopped with 2N H_2_SO_4_. Absorbance values were determined at 450 nm using the Multiskan EX reader (Thermo Scientific).

#### 3.11.7. Alkaline Phosphate (ALP) Activity

The ALP activity was measured in previously obtained cell lysates by a p-NPP (para-nitrophenylphosphate) hydrolysis assay. Then 100 µL p-NPP (4 µg/µL) were added to 100-µL samples in a 96-well plate and incubated at 37 °C for 30 min. Then, a 2 M NaOH solution was added to stop the reaction. The optical density at 405 nm was determined using the Multiskan EX reader (Thermo Scientific). The standard curve (range: 0 to 10 IU/mL) of ALP (Molecular Biology, Thermo Scientific) was used to calculate the enzyme activity in the samples.

#### 3.11.8. Statistical Analysis

Intergroup outcomes were compared for statistical significance using a one-way ANOVA (analysis of variance). In all cases, significance was accepted at *p* < 0.05. All analyses were performed using GraphPad Prism 7 software (GraphPad Software, San Diego, CA, USA).

## 4. Conclusions

In this study, we successfully prepared a ternary foam scaffold using PCL, hydroXyapatite, and L-Lysine (50/48/2 wt% ratio). The scaffold had a ~45% higher Young’s modulus than the PCL/HAP (50/50 wt% ratio) reference sample and porosity at a level of over 90%. The incorporation of L-Lysine as a filler causes a significant improvement of the compressive stress compared to the PCL and PCL/HAP systems. At the same time, the contact angle decreased from 88° for PCL to 78° for PCL/HAP and 73° for PCL/HAP/Lys. It was found that L-Lysine was released from the PCL/HAP/Lys scaffold within 20 h. We have shown that PCL and PCL-modified composites enable cell growth and proliferation. Moreover, the bone biocompatibility of PCL scaffolds can be improved by adding hydroxyapatite and/or L-Lysine. It is worth emphasizing that, in our research, the modification of PCL/HAP scaffold with L-Lysine resulted in the significant intensification of pre-osteoblasts’ proliferation and supported sequential differentiation, as judged by IL-6, IL-1β, and alkaline phosphatase production by the cells. The modification of scaffolds with HAP and L-Lysine resulted in efficient scaffold colonization, which in turn should translate to high-density bone tissue formation and the enhanced regenerative potential of such composites. It is known that the coating of material surfaces with L-Lysine provides cationic site, which lead to increased adhesion and improved cytobiocompatibility. Overall, the biological assessment of the PCL/HAP/Lys scaffolds points to them as promising candidates for future biomedical engineering applications.

## Figures and Tables

**Table 1:** Density and porosity of PLC-based porous scaffolds.

Sample	Density, $\rho_{sc}$ (g·cm^−3^) (10^−2^)	Bulk Density (10^−2^)	Porosity (%)
PCL	7.0 ± 0.2	10.9 ± 0.2	93.6 ± 0.2
PCL/HAP	11.0 ± 0.4	12.8 ± 0.3	91.4 ± 0.2
PCL/HAP/Lys	11.6 ± 0.3	12.3 ± 0.1	90.6 ± 0.3

**Table 2:** Compressive strength measurements of PLC-based porous scaffolds.

Sample	Compressive Stress at 40% Strain (kPa)	Compressive Stress at 80% Strain (kPa)	Young’s Modulus (kPa)
PCL	49.7 ± 5.6	326.8 ± 24.7	204.6 ± 19.4
PCL/HAP	47.6 ± 4.3	424.3 ± 39.1	318.2 ± 22.1
PCL/HAP/Lys	80.2± 9.6	527.6 ± 44.1	464.4 ± 26.9

**Table 3:** The values of water contact angles and water uptake for PCL-based porous scaffolds.

Sample	Water Contact Angle, θ (°)	Water Uptake, W.U. (%)
PCL	87.8 ± 3.0	1192 ± 3.1
PCL/HAP	77.9 ± 4.5	835 ± 39.7
PCL/HAP/Lys	72.6 ± 4.2	735 ± 17.4

**Table 4:** Mass loss at 900 °C and the temperature corresponding to 5 wt% loss of mass for PCL-based porous scaffolds.

Sample	Mass Loss at 900 °C, (%)	HAP Content, (wt%)	T_–5 wt%_ (°C)	Inflection Point
PCL	99.06		381.6	425.9
PCL/HAP	51.81	48.19	378.2	421.6
PCL/HAP/Lys	52.84	47.16	380.2	421.6
HAP	3.93	100		
LYS	96.57		221.7	

**Table 5:** Thermal parameters from the cooling and second heating DSC curves of PCL-based porous scaffolds.

Sample	T_c_^onset^ (°C)	T_c_ (°C)	ΔH_c_ (J/g)	T_m_^onset^ (°C)	T_m_ (°C)	ΔH_m_ (J/g)	X_c_ (%)
PCL	37.3	32.5	−55.8	53.7	58.3	55.5	39.9
PCL/HAP	37.0	33.2	−27.3	53.1	59.2	28.4	40.8
PCL/HAP/Lys	38.5	35.6	−27.3	54.0	59.2	27.7	39.9

**Table 6:** List and designation of the samples investigated in the study.

Sample	Apatite Whiskers Content (wt%)	L-Lysine Content (wt%)
PCL	-	-
PCL/HAP	50	-
PCL/HAP/Lys	48	2

## Data Availability

Data are contained within the article.
